# Atlanta Obstetrical Society

**Published:** 1894-10

**Authors:** 


### ATLANTA OBSTETRICAL SOCIETY.

ATLANTA, July 12, 1894.

Dr. V. O. Hardon made some remarks on the "Treatment of Puerperal Peritonitis." (See page 449.)

### DISCUSSION.

DR. CROW: I have been very much interested in this subject for several years, and can say that I agree, on the whole, with the speaker in the management of these cases. There is not the slightest question in my mind as to the advisability of operating in a number of these cases. The question to decide is when to operate, for we all know that a great many cases of peritonitis of a violent type will get well under proper medicinal treatment. Still there is a large per cent. of these cases of peritonitis that we have from time to time that do not respond in the least to our treatment, but steadily grow worse and die, if not relieved by the only means left us,—that is, by operative interference. I was called to see a case some three weeks ago with Dr. J. C. Avary, that demonstrates to my mind the absolute necessity of operating in these extreme cases. This patient had been confined some six weeks previous, had had some trouble since her confinement, but not of a serious character. On the morning of my seeing her she was taken with a severe pain in lower portion of abdomen which was followed by collapse and extreme prostration; the family physician injected stimulants and applied hot bottles to extremities. Dr. Avary and I saw her about two o'clock P. M., and decided to open the abdomen at once. Her pulse was over 150 beats per minute, and it seemed very much as if she would die on the table. The abdomen was filled with bloody serum. There was an extensive exudate on the left side. We thoroughly washed out the abdomen with plain hot water, temperature 110°, then closed the incision with a drainage tube in lower angle. Her pulse was better when she was put in bed than when she was put on the table. I am confident that we saved her life by the only possible means at our command. In cases of septic peritonitis I always give tr. ferri. chlor. in good doses, with a glycerin menstruum in connection with the other treatment as spoken of here to-night.

DR. KIME: When we come to the question of infection it concerns both the specialist and general practitioner, the specialist seeing many cases in consultation when it requires active measures. The treatment is based on three cardinal principles: *cleansing, purgation,* and *drainage*. Clear uterine cavity, render it aseptic, and try to keep it so by irrigation, disinfection, and drainage. The rubber drainage tube is of benefit in these cases if properly used. To these add good liquid nourishment, tonics, including stimulation and strychnia. If these fail operative measures should be considered. In the cases designated by the speaker as primary general peritonitis, my views as to operation are in accord with his. Where the uterine muscular structures, tubes, or ovaries are involved, then opening up and washing out will not relieve, for foci of infection are left behind. I was in hopes the doctor would enter deeper into the details of indications for operative measures in these cases.

DR. BAIRD said he had been very much interested in the remarks of Dr. Hardon. Reference had repeatedly been made by the speaker to "infective peritonitis." Is it only to one form of peritonitis that he considers the operation which he advocates applicable? If so, how does he differentiate this particular form from other forms? What are the trustworthy diagnostic signs and symptoms? If operative measures are practiced only as a *dernier ressort* of course a differential diagnosis is not so important.

In his experience he had been singularly fortunate in freedom from septic infection in his puerperal cases. This immunity from septic complications he attributes largely to the extraordinay care which he invariably takes to secure firm and permanent contraction of the uterus before leaving his patient. This contraction he considers the very best possible antiseptic. He relies upon it, supplemented by cleanliness, almost exclusively, as he never uses either vaginal or uterine douches without some distinct indication for their employment.

DR. HARDON: As to what should be done in cases presenting diseased tubes, foci of pus in uterus, etc., with the consent of relatives after opening abdomen would remove diseased organs. If there be disease of pelvic organs, would first have evidence of disease in these organs before manifestations are made that others are affected. Bimanual vaginal examination will reveal focus. Have never had a case of general peritonitis result from pelvic peritonitis. Am inclined to think that cases starting as pelvic peritonitis and becoming general do not show the same serious evidences as those beginning as general peritonitis. In last case operated upon shock was somewhat relieved by the operation. When begun, pulse was 148 and weak; in an incredibly short time after the irrigation with hot water began pulse dropped to 118, and became much stronger. This improvement continued until the patient was out of danger.

---

### ANTIQUITY OF MEDICAL DEGREES.

The celebration five years ago of the eighth century of the founding of the University of Bologna gave fresh prominence to the fact that she is not only the mother of seats of learning, but also of academic degrees. It was in the faculty of law that she first conferred the honor of graduation, Doctor Legum being the first title to which the regenerator of the Roman juridical system, and the virtual founder of the Bologna school, promoted the alumnus who had attended the prescribed courses and passed the qualifying ordeal.

Paris still in the faculty of law, imitated the example of her Italian sister, whilst not till a century later did England follow suit. Medicine having, like law, assumed the dignity of a faculty, began also to give the title of doctor, a word which is met with in that connection as early as the first century, when Suetonius relates of Julius Cæsar that he invested with the rights of Roman citizens, "*omnes medicinam professos et liberalium artium doctores.*" From the earliest time the practitioner of an art, particularly that of medicine, was looked up to as its teacher. And so when the healing art, incorporated in the university system, handed on the torch of 'light and leading' to its disciples, it did so by promoting them from alumni to be doctors, equally qualified to practice and to instruct."—*London Lancet.*

